# Ambulatory assessment of phonotraumatic vocal hyperfunction using glottal airflow measures estimated from neck-surface acceleration

**DOI:** 10.1371/journal.pone.0209017

**Published:** 2018-12-20

**Authors:** Juan P. Cortés, Víctor M. Espinoza, Marzyeh Ghassemi, Daryush D. Mehta, Jarrad H. Van Stan, Robert E. Hillman, John V. Guttag, Matías Zañartu

**Affiliations:** 1 Department of Electronic Engineering, Universidad Técnica Federico Santa María, Valparaíso, Chile; 2 Department of Sound, Universidad de Chile, Santiago, Chile; 3 Computer Science and Artificial Intelligence Laboratory, Massachusetts Institute of Technology, Cambridge, MA, United States of America; 4 Center for Laryngeal Surgery and Voice Rehabilitation, and MGH Institute of Health Professions, Massachusetts General Hospital, Boston, MA, United States of America; 5 Department of Surgery, Harvard Medical School, Boston, MA, United States of America; Griffith University, AUSTRALIA

## Abstract

Phonotraumatic vocal hyperfunction (PVH) is associated with chronic misuse and/or abuse of voice that can result in lesions such as vocal fold nodules. The clinical aerodynamic assessment of vocal function has been recently shown to differentiate between patients with PVH and healthy controls to provide meaningful insight into pathophysiological mechanisms associated with these disorders. However, all current clinical assessment of PVH is incomplete because of its inability to objectively identify the type and extent of detrimental phonatory function that is associated with PVH during daily voice use. The current study sought to address this issue by incorporating, for the first time in a comprehensive ambulatory assessment, glottal airflow parameters estimated from a neck-mounted accelerometer and recorded to a smartphone-based voice monitor. We tested this approach on 48 patients with vocal fold nodules and 48 matched healthy-control subjects who each wore the voice monitor for a week. Seven glottal airflow features were estimated every 50 ms using an impedance-based inverse filtering scheme, and seven high-order summary statistics of each feature were computed every 5 minutes over voiced segments. Based on a univariate hypothesis testing, eight glottal airflow summary statistics were found to be statistically different between patient and healthy-control groups. *L*_1_-regularized logistic regression for a supervised classification task yielded a mean (standard deviation) area under the ROC curve of 0.82 (0.25) and an accuracy of 0.83 (0.14). These results outperform the state-of-the-art classification for the same classification task and provide a new avenue to improve the assessment and treatment of hyperfunctional voice disorders.

## Introduction

Voice disorders affect approximately 6.6% of the working population in the United States [1] and can have devastating psychological and social-economic consequences on those impacted. The most common voice disorders are chronic or recurring conditions that are believed to be caused by detrimental patterns of vocal behavior, referred to as vocal hyperfunction [2]. Such behaviors are often associated with trauma-induced lesions of the vocal folds (e.g., nodules, polyps), which we refer to as phonotraumatic vocal hyperfunction (PVH) [3]. Despite the significance prevalence of hyperfunctional voice problems, effective prevention and clinical management continues to be hampered by limited knowledge of the etiological and pathophysiological mechanisms related to these disorders. For example, even though daily voice use is often assumed to be a critical factor, the actual relationships between daily voice use and vocal hyperfunction is not well understood.

There have been some recent attempts to better characterize hyperfunctional voice disorders. In an expansion of previous work [2], it has been more definitively demonstrated that glottal aerodynamic measures of subglottal air pressure, and glottal airflow (normalized by sound pressure level) can be used to identify phonatory mechanisms associated with vocal hyperfunction that are distinctly different from normal vocal function [4]. These glottal airflow measures were obtained in the laboratory using a circumferentially vented (CV) pneumotachograph mask to capture oral airflow with a bandwidth of approximately 0 Hz to 1.2 kHz [5]. The oral airflow waveform was then inverse filtered (e.g., [2], [6], [7], [8]) to remove the influence of the vocal tract, and thus estimate clinically parameters of the glottal airflow waveform, such as peak-to-peak AC flow (ACFL), open quotient (OQ), and maximum flow declination rate (MFDR). In terms of clinically interpretability, the works of [2] and [4] provide a robust framework for which aerodynamic measures are useful to differentiate vocal hyperfunction from normal voice.

The aerodynamic-based differentiation between normal vocal function and pathophysiological mechanisms of PVH has been further supported and elucidated in recent investigations employing computer modeling. In particular, these studies have demonstrated that the elevation of ACFL and MFDR can be associated with the compensation that is necessary for individuals with PVH to maintain normal loudness [9], [10] in the presence of vocal fold pathology. This compensatory behavior contributes to what has been described clinically as a “vicious cycle” of continued concomitant increases/worsening of phonotrauma and PVH. Such compensation presents additional challenges in attempting to identify purely etiological factors. The present work focuses only on PVH subjects with the mentioned compensatory behavior, and not necessarily discriminates subjects with other characteristics of vocal fold pathologies, such as for example incomplete glottal closure. Work related to the analysis of aerodynamic measures for normal subjects and subjects with unilateral vocal fold paralysis can be found in [11] and [12]

**Fig 1:**
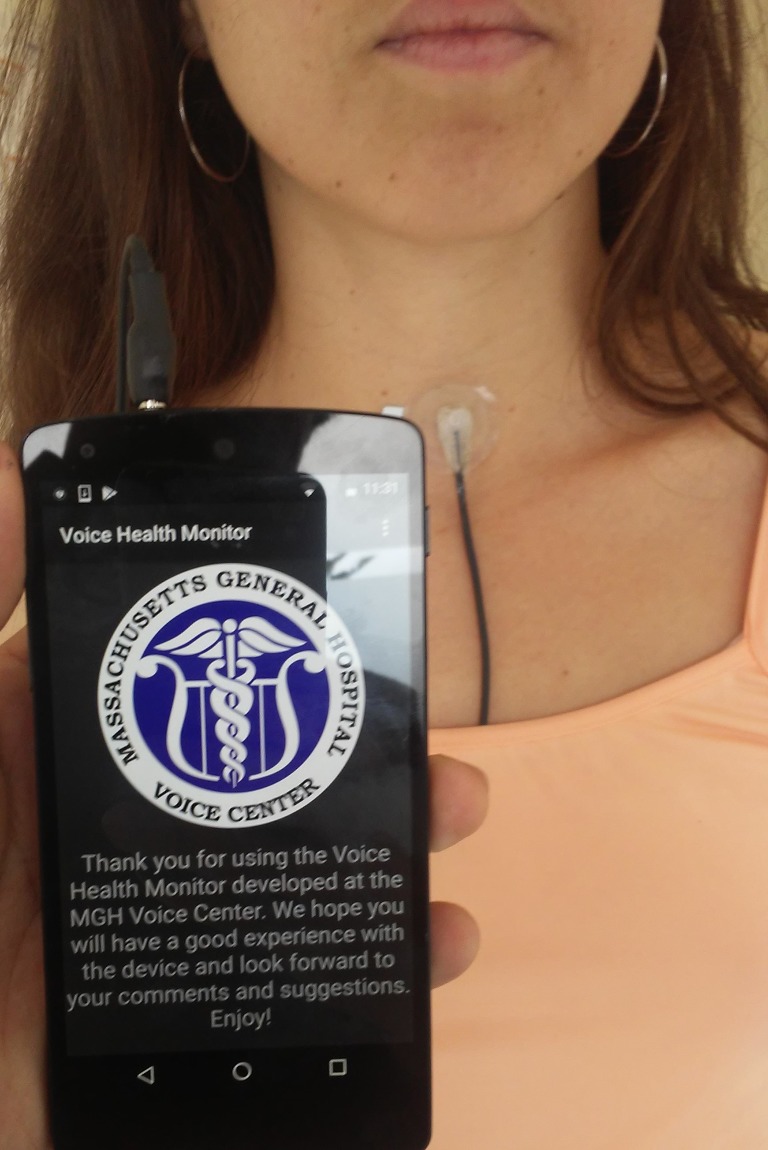
Example of VHM system. Illustration of the smartphone-based ambulatory voice monitor that uses a neck-surface accelerometer attached to the skin halfway between the thyroid prominence and the suprasternal notch of a female subject.

Ambulatory voice monitoring technology has been developed over several decades to investigate daily voice use. Our group has developed a smartphone-based ambulatory voice monitor (see Fig 1) that uses an application to capture and store the high-bandwidth signal from a light-weight accelerometer (ACC) attached to the front of the neck below the thyroid prominence and can be comfortably worn for multiple days at a time [3], [13]. Measures typically extracted from the voice monitor recordings are based on estimates of sound pressure, level, (SPL), fundamental frequency, and voicing duration, including cumulative vocal dose parameters such as phonation time, cycle dose, and distance dose [14]. Univariate statistical analysis of long-term data from individuals with PVH and matched healthy-controls have not shown the expected differences between overall average measures of voice use (i.e., PVH subjects did not, on average, talk more or louder than healthy controls), which suggests that such measures may not be directly useful clinically in helping to identify relevant aberrant vocal behaviors [15]. However, using features derived from these measures (mostly higher-order distribution-based statistics) in a supervised classification task demonstrated statistically significant differentiation between individuals with PVH and healthy controls, with an area under the ROC curve (AUC) of 0.705 and F-score of 0.630 for a small dataset [16]. Analysis of a larger dataset with 102 subjects (51 patient-control pairs) resulted in an AUC of 0.739 and F-score of 0.766 [3]. While promising, these findings may not be readily translated for clinical use because the level of performance may still not be adequate (marginal ability to differentiate between normal and disordered subjects), and because the resulting features do not provide direct insights into underlying pathophysiological mechanisms associated with vocal hyperfunction—i.e. the features are based on measures extracted from the voice acoustic output signal which cannot provide information about the specific physiologic parameters/mechanisms that produce voice (e.g. glottal volume velocity source characteristics). Similar limitations are observed in recent deep learning approaches [17], that still lack physiological and clinical relevance since they operate in sustained vowel scenarios and do not provide additional insights for voice therapy or biofeedback. Further efforts are need to advance ambulatory monitoring of voice with physiologically relevant features that can help to identify vocal hyperfunction.

In this study, we investigate whether ambulatory estimates of glottal airflow parameters can significantly differentiate between normal vocal activity and activity associated with PVH. This is the first analysis of ambulatory estimation and assessment of aerodynamic measures using a large group of PVH subjects. There is evidence in physical models [9] and real subjects [4] that PVH behavior manifests by compensation of SPL by producing higher levels of ACFL than normal voice function. Recognizing these features could improve clinical assessment of PVH by combining the advantages of glottal airflow measures and ambulatory monitoring. To accomplish this task, we used and extended an impedance-based inverse filtering (IBIF) scheme to estimate the high-bandwidth glottal airflow waveform from the neck-surface ACC signal [18].

This is the first effort to advance the IBIF algorithm into an ambulatory scenario, as the original study [18] only used sustained vowels and laboratory conditions. Thus, some additional considerations and details for the IBIF scheme are provided for this purpose. Note that Mehta et. al. [3] plotted the distribution of MFDR for a week for a single subject as proof of concept that IBIF could be potentially used to extract aerodynamic features. However, no quantitative analysis was performed for that case study.

## Subglottal impedance based inverse filtering for ambulatory monitoring of voice

**Fig 2:**
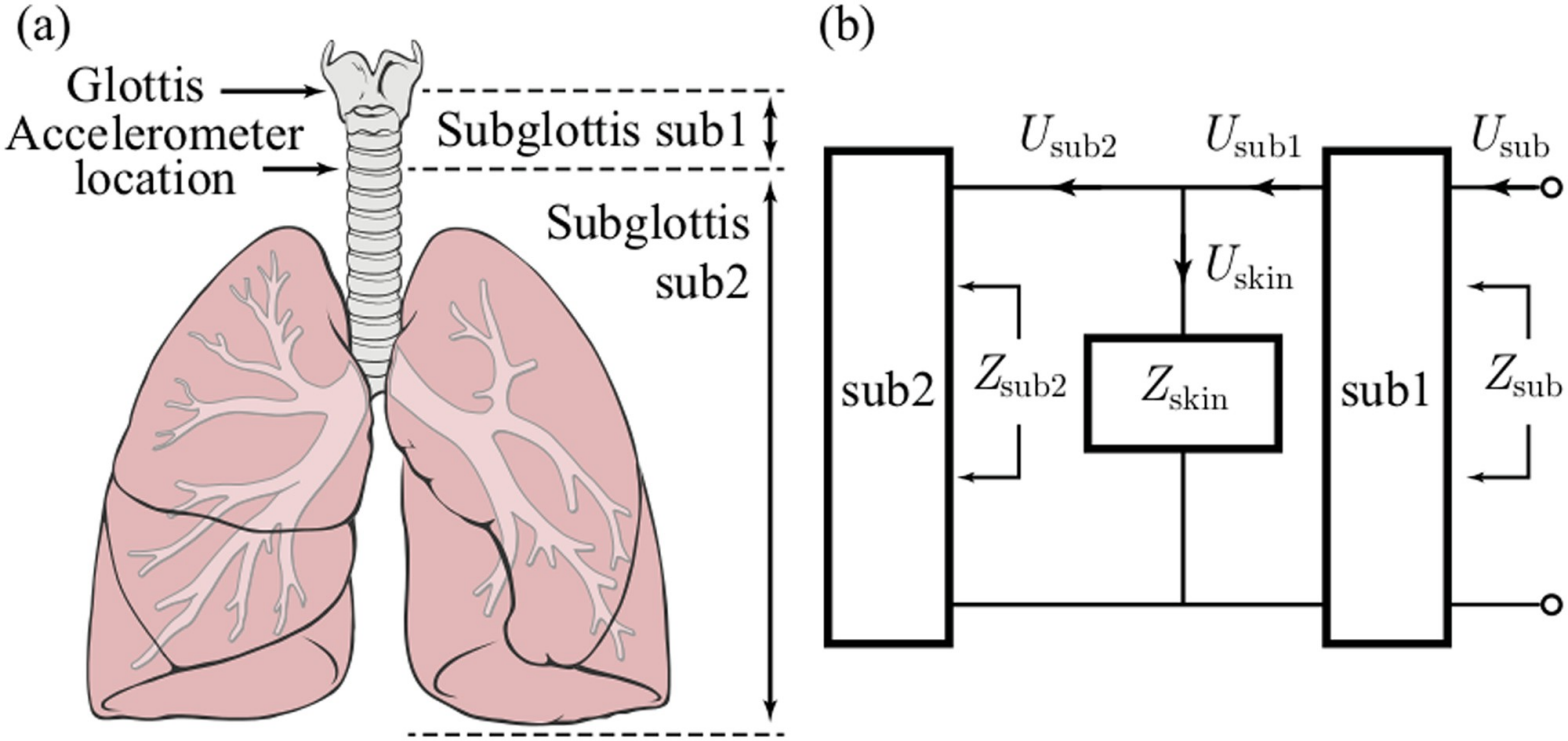
Representation of the subglottal system. (a) Accelerometer position and *sub1* and *sub2* system parts. (b) A mechano-acoustic analogy of the subglottal system including load impedance from skin. Reproduced with permission.

In this section, the IBIF algorithm [18] is summarized but also extended and optimized for ambulatory voice monitoring. The IBIF is a model-based scheme to estimate the glottal airflow from neck-surface acceleration [18]. The method uses a mechano-acoustic transmission line model to account for the acoustic propagation in the subglottal system and neck skin characteristics. The scheme is illustrated in Fig 2, where the electrical equivalent circuit shows the interconnection between the subglottal tracts above and below the location of the accelerometer (sub1 and sub2, respectively) and load impedance of the skin *Z*_*skin*_, that also includes the radiation load of the accelerometer sensor *Z*_*rad*_. The glottal airflow signal estimate ${\hat{u}}_{g}\left(t\right)$ to be obtained from the accelerometer signal ${\dot{u}}_{skin}\left(t\right)$ is calculated using Eq (1):

$$\begin{array}{c} {\hat{u}}_{g}\left(t\right)=F^{-1}(-\frac{{\dot{U}}_{skin}\left(\omega \right)\cdot A_{acc}}{T_{skin}\left(\omega \right)}), \end{array}$$ (1)

with

$$T_{skin}\left(\omega \right)=\frac{H_{sub1}\left(\omega \right)\cdot Z_{sub2}\left(\omega \right)\cdot j\omega }{Z_{sub2}\left(\omega \right)+Z_{skin}\left(\omega \right)},$$ (2)

$$Z_{skin}\left(\omega \right)=\frac{1}{A_{acc}}\{R_{m}+j\omega M_{m}-\frac{j}{\omega }K_{m}+Z_{rad}\left(\omega \right)\},$$ (3)

$$Z_{rad}\left(\omega \right)=\frac{j\omega \cdot M_{acc}}{A_{acc}},$$ (4)

where $F^{-1}(\cdot )$ is the inverse Fourier transform, *H*_*sub*1_(*ω*) = *U*_*sub*1_(*ω*)/*U*_*sub*_(*ω*) is the transfer function of subglottal section *sub1* (see Fig 2), *A*_*acc*_ the accelerometer area (cm^2^), *M*_*acc*_ the accelerometer mass (gr), and ${\dot{U}}_{skin}\left(\omega \right)$ is the acceleration signal in frequency domain. *Z*_*sub*2_ and *H*_*sub*1_ are calculated using an anatomically based, acoustic model of the subglottal system [18–20]. *Z*_*rad*_ corresponds to the radiation impedance from the accelerometer. All frequency and time-domain expressions are sampled and processed appropriately [21].

In order to use IBIF as a signal processing tool, subject-specific parameters need to be estimated. These IBIF parameters are scaling factors that adjust default values of the mechanical impedance model of neck skin surface, length of the trachea, and accelerometer location. The parameters are represented in a set **Q** = {*Q*_*i*_}_*i*=1,…,5_ for neck skin resistance *R*_*m*_, mass *M*_*m*_, and stiffness *K*_*m*_, as well as length of the trachea *L*_*trachea*_ and accelerometer placement *L*_*sub*1_. Each of these Q parameters is bounded to maintain physiological plausibility [18]. The magnitude terms in Eq (3) are the default values for each parameter [22], which are scaled for normalized Q factor as, *R*_*m*_ = 2320 ⋅ *Q*_1_ in (g ⋅ s^−1^ ⋅ cm^−2^), *M*_*m*_ = 2.4 ⋅ *Q*_2_ in (g ⋅ cm^−2^), *K*_*m*_ = 491000 ⋅ *Q*_3_ in (dyn ⋅ cm^−3^), and for *Z*_*sub*2_(*ω*), *L*_*trachea*_ = 10 ⋅ *Q*_4_, and *L*_*sub*1_ = 5 ⋅ *Q*_5_ are in (cm). Note that default model parameter are obtained for **Q** = [1, 1, 1, 1, 1] [18]. Using these subject-specific factors will allow to filter out neck-skin and subglottal resonances, making the estimated glottal airflow signals comparable between subjects.

To obtain subject-specific IBIF parameters, we compare the IBIF-derived glottal airflow waveform estimates with that from the current gold standard, namely an inverse filtered glottal airflow signal obtained from recordings using a CV pneumotachograph mask [5]. Inverse filtering in this case is a challenging task given the reduced bandwidth of the CV mask due to the airflow transducers (PT-2E, Glottal Enterprises) and the type of voices that will be analyzed (high-pitched female voices exhibiting pathology). Inverse filtering of the oral airflow was performed using a semi-automatic approach, as recently described in [4]. This approach was particularly designed to inverse filter normal and pathological high-pitched voices from a CV mask signal.

Once we obtain an estimate of the glottal airflow from the CV mask, we run a Particle Swarm Optimization (PSO) scheme [23], which consists in the optimization of a non-linear continuous fitness function thorough the search of optimal “particles” (parameters) by searching its best set. For this case, PSO searches the optimal Q parameters that represent the subject’s anatomical features. The fitness function in this optimization process needs to yield robust and consistent solutions. We minimize the following normalized weighted absolute error (NWAE) function, such that

$$\begin{array}{c} \text{NWAE}\left(Q\right)=\sum_{i=1}^{3}w_{i}\cdot e_{i}\left(Q\right), \end{array}$$ (5)

with

$$\begin{array}{c} \;\sum_{i=1}^{3}w_{i}=1,\;0\leq w_{i}\leq 1, \end{array}$$ (6)

and

$$\begin{array}{c} e_{i}\left(Q\right)=\frac{\sum_{n=0}^{N-1}|\Delta^{\left(i-1\right)}{\tilde{u}}_{g}-\Delta^{\left(i-1\right)}{\hat{u}}_{g}|}{\sum_{n=0}^{N-1}|\Delta^{\left(i-1\right)}{\tilde{u}}_{g}|}, \end{array}$$ (7)

where ${\tilde{u}}_{g}$ is the CV mask-based inverse-filtered glottal airflow signal, ${\hat{u}}_{g}$ is a time-aligned IBIF-based glottal airflow signal, Δ^(*i*−1)^ the time-derivative operator of order (*i* − 1), and *i* represents the index of the corresponding error function *e*_*i*_ and its weight *w*_*i*_. Each weighting *w*_*i*_ was set to $0.\bar{3}$. The increased order of the time-derivative operator is used to balance the energy of higher harmonics in NWAE to avoid over-fitting in the low frequency range. Therefore, the optimization problem is stated as:

$$\begin{array}{c} \hat{Q}=\text{arg}\;\underset{Q}{\text{min}}\text{NWAE}\left(Q\right),\;\text{subject}\;\text{to}\;Q\in D, \end{array}$$ (8)

where **D** = {*D*_*i*_}_*i*=1,…,5_, is a set of restrictions for each parameter within the **Q** set that is designed to maintain physiological plausibility [18]. To reduce the computational load of PSO, several configurations of subglottal systems were pre-calculated (i.e., before the PSO algorithm started) for a set of equally spaced values of tracheal length and accelerometer position. Each pre-calculated (*Z*_*sub*_ and *H*_*sub*1_) transfer function was indexed and retrieved inside the PSO algorithm. This approach substantially reduces the computational time of the optimization process.

The time-alignment of the oral airflow and acceleration signals is as follows. A first approximation is to align using the sample cross-correlation function [21] and find the maximum peak shifted in the neighborhood of mid-lag position [24]. To improve this initial approximation, a delay parameter *d* is added in the PSO algorithm by shifting the indices of signal vectors (oral airflow and neck acceleration). Since the shifted signal (oral airflow) is delayed for only a few samples, the search space is limited to *d* ∈ *D*_0_ = [−*d*_0_, *d*_0_] where *d*_0_ is a small number ∈ **Z**^+^. Then, given *N*(≫ *d*_0_) samples of data, ${\tilde{u}}_{g}$ and ${\hat{u}}_{g}$ are replaced in (7) by

$$\begin{array}{cc} {\hat{u}}_{gt}\left(nT\right)\; & ;\;n\in [d_{0},N-1-d_{0}],\;\text{and} \end{array}$$ (9)

$$\begin{array}{cc} {\tilde{u}}_{gtd}\left(nT\right)\; & ;\;n\in [d_{0}+d,N-1-d_{0}+d]. \end{array}$$ (10)

Note that ${\hat{u}}_{gt}\left(nT\right)$ is a trimmed version of ${\hat{u}}_{g}\left(nT\right)$ and ${\tilde{u}}_{gtd}\left(nT\right)$ is a trimmed, delayed version of ${\tilde{u}}_{g}\left(nT\right)$ both with *N* − 2*d*_0_ samples, where *T* is the sampling period. An initial value for *d*_0_ was half the average glottal cycle duration.

In the case of incomplete glottal closure, coupling between the subglottal tract and vocal tract is embedded in the resulting dipole source [25]. Therefore, the glottal flow with all the source-filter interactions can be estimated without the need to model glottal coupling.

## Methods

### Experimental setup and participants

The human studies protocol used to collect the data for this study (Ambulatory monitoring of vocal function to improve voice disorder assessment: #2011P002376) was approved by the Institutional Review of the Partners Healthcare System—the Massachusetts General Hospital is a founding member of this organization. Dr. Robert E. Hillman is the PI on this protocol. Study participants were 48 pairs of adult females (total of 96 subjects) with each pair comprised of one patient with PVH (diagnosed with vocal nodules) and one normal control subject matched to the patient by age and occupation (see Table 1 for more details). Diagnoses were based on a complete team evaluation by laryngologists and speech-language pathologists at the Massachusetts General Hospital Voice Center that included (a) a complete case history, (b) endoscopic imaging of the larynx, (c) aerodynamic and acoustic assessment of vocal function based on Mehta et. al. [26], (d) a patient-reported Voice-Related Quality of Life questionnaire, and (e) a clinician-administered Consensus Auditory-Perceptual Evaluation of Voice assessment (CAPE-V). All patients were enrolled prior to the administration of any voice treatment. Written informed consent was obtained from all subjects. All subjects were 18 years of age or older. Due to the higher incidence of female patients with PVH than men in the overall population [27], only women were subjects for this study. Zhukhovitskaya et. al. [28] have shown significant differences (*p* < 0.0001) in the number of bilateral midfold lesions between males and women. Moreover, the inclusion of men would create confounding variables due to sex-specific characteristics. The matching is done to normalize for general vocal behavior differences. For example, males and females have anatomical differences, there are voice changes with age (for example, presbyphonia usually occurs when people gets older), and the type of occupation is related to how much voicing is used during a typical day at work. On the other hand, the subject-specific parameters from IBIF are normalized for each individual, so signals can be comparable, due to differences in neck-skin and subglottal anatomy. Therefore, these are not matched on healthy-patient pairs.

**Table 1:** Occupations and mean age of adult females with PVH and matched-control participants analyzed (48 pairs).

Occupation	No. subject pairs	Age ^a^	Diagnosis	CAPE-V overall ^b^
**Singer**	34	21.3 (3.7)	Nodules (31) Polyp (3)	21.2 (12.6)
**Teacher**	5	38.9 (12.1)	Nodules	33.8 (18.8)
**Consultant**	2	23 (1.4)	Nodules (1) Polyp (1)	22.0 (5.7)
**Psychologist**	1	34 (P) 30 (C)	Nodules	−
**Recruiter**	2	23.5 (0.8)	Nodules	40.5 (13.4)
**Marketer**	1	22 (P) 25 (C)	Nodules	25
**Media relations**	1	32 (P) 31 (C)	Nodules	30
**Registered nurse**	1	57 (P) 58 (C)	Polyp	40

^*a*^Mean age and (standard deviation) are shown for pairs ≥ 2. Otherwise, the age is shown for the phonotraumatic (P) and control (C) subject.

^*b*^Mean overall severity score (0-100) and (standard deviation) are shown patients from pairs ≥ 2. Otherwise, the patient’s score is shown.

Each subject was recorded as they engaged in normal daily activities during one week using the smartphone-based ambulatory voice monitor [3, 13]. The system employs an accelerometer attached to the front of the neck below the larynx as the phonation sensor (see Fig 1). The sampling frequency was 11,025 Hz and the average total recording time for a subject was approximately 80 hours, as in [15] [3].

Each subject underwent a session in the laboratory to obtain a subject-specific calibration for the IBIF algorithm. The session involved simultaneous and synchronous recordings of CV mask-based oral airflow and neck skin acceleration in an acoustically treated room. Each subject performed a series of sustained vowels gestures (/a/ and /i/) with a constant pitch using comfortable and loud (approximately 6 dB increase) voice. For each gesture, a bandpass filter (60 − 1100 Hz) oral airflow vowel segment was used to perform inverse filtering with a single notch filter constrained to unitary gain at DC [29].

Once a glottal airflow approximation is obtained from the CV mask, Q parameters are estimated using the optimization scheme described in the *Subglottal Impedance Based Inverse Filtering for Ambulatory Monitoring of Voice* section. The whole process, from estimation of parameters to classification and statistical analysis was done with MATLAB (The MathWorks, Inc.).

### Ambulatory glottal airflow assessment

Estimates of individual Q parameters, which were assumed to be time-invariant for each subject, were applied in Eq (1). The assumption of time-invariance is due to the properties of the neck skin, which should not change over time. Preliminary studies of the use of IBIF calibrated for a single vowel [30] and on the variability of these calibrated parameters [31], have shown that using a sustained vowel works well on running speech (e.g., the rainbow passage). Current research aims to explain in more detail the estimation and variability of these parameters under different speech conditions. ${\dot{u}}_{skin}\left(k\right)$ the discrete time-domain equivalent of the acceleration signal ${\dot{U}}_{skin}\left(\omega \right)$, is convolved with *t*_*skin*_(*k*) the inverse transfer function of the skin in time domain, where its frequency domain expression is represented by Eq 2.

By taking the inverse fast Fourier transform (IFFT) with 1102 coefficients, we obtain *t*_*skin*_(*k*), a FIR filter. We take every consecutive hour of the acceleration signal ${\dot{u}}_{skin}\left(k\right)$ and convolved it with *t*_*skin*_(*k*) to obtain the estimated glottal flow signal ${\hat{u}}_{g}\left(k\right)$. This signal was segmented into 50 ms non-overlapping windows. Voiced frames from the ACC signal were identified based on the same voice activity detection algorithm used in [3], where a combination of periodicity and spectral metrics whether a frame is voiced or unvoiced. In addition, we discarded frames in which the absolute ratio of the RMS values of the first half divided by the second half of the frame was greater than a threshold (1.5); thus, frames exhibiting onsets or offsets were removed since they typically result in incorrect inverse filtering estimates due to cycle-by-cycle variations in the signal. As with many inverse filtering methods [32], IBIF has difficulty analyzing signal with high *f*_0_ values due to the short closed phase during which vocal tract information must be estimated (females and singers, especially, produce high-pitched phonation). Performance of traditional glottal inverse methods could be accurate up to a *f*_0_ of 400 Hz [33]. By visual inspection, the estimation of IBIF voiced frames deteriorated around a *f*_0_ of 500 Hz. Thus, voiced frames with *f*_0_ higher than 500 Hz were not processed by IBIF. Future research will analyze sensitivity tests to find the range of frequencies for which the IBIF method fails.

**Fig 3:**
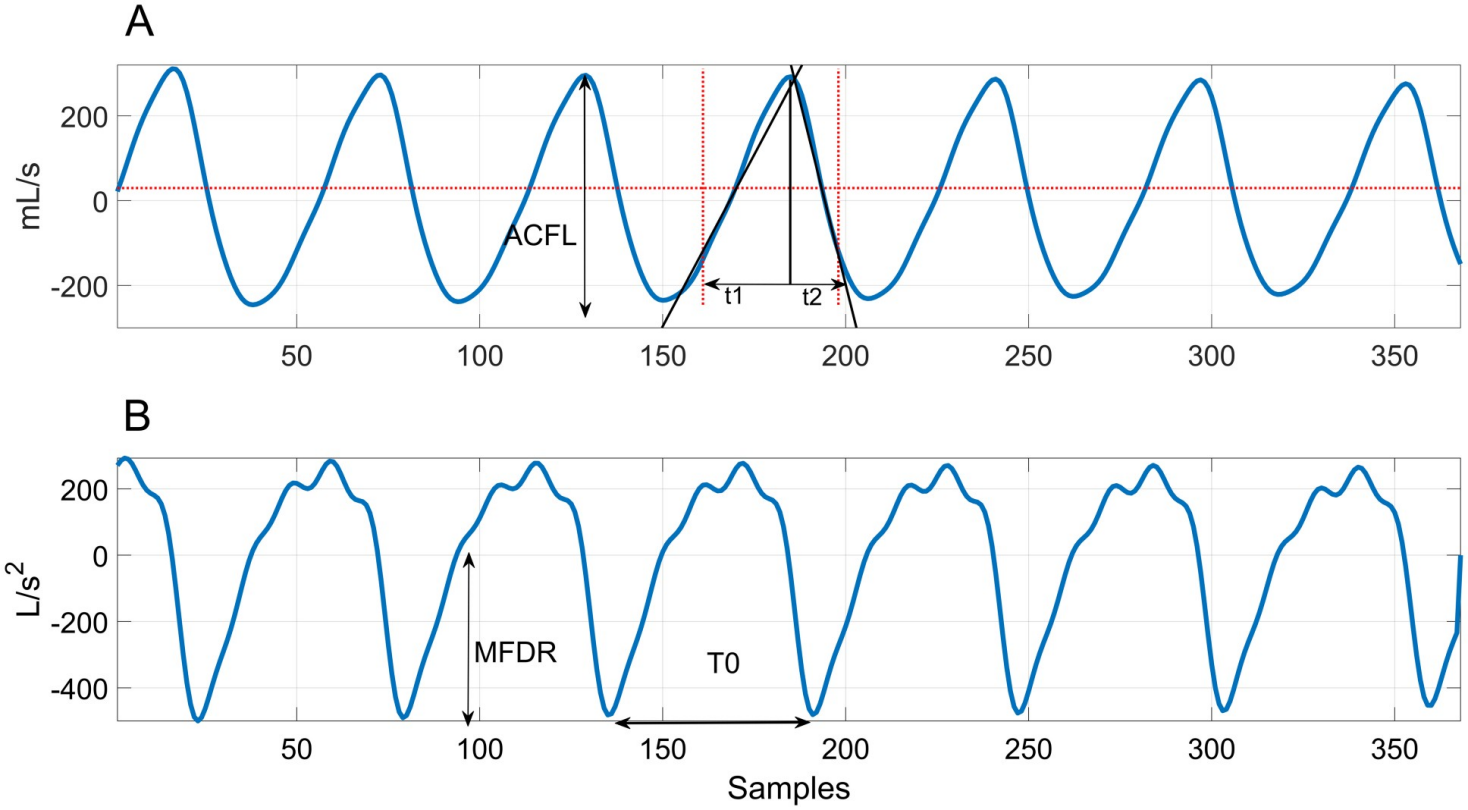
Example of ambulatory IBIF analysis. (A) Estimated glottal airflow waveform and (B) its derivative, showing how time-domain measures were derived per glottal cycle. Measures were then averaged over all cycles to yield a single value per frame for each time-domain measure.

Table 2 lists the 11 glottal airflow measures computed within each analyzed frame. Fig 3 shows an example of the estimated glottal airflow signal and its derivative for a single frame. Since the accelerometer is an AC signal, the glottal airflow does not have a DC component. As in previous studies [2, 4, 34], ACFL was obtained as the difference between the maximum and minimum amplitude (peak-to-peak) within each glottal cycle. MFDR was the minimum value of the derivative of one glottal cycle. For open and speed quotient, the closed phase in ambulatory settings often exhibits more fluctuations than in laboratory conditions using sustained vowels. For robust estimations of open and speed quotient, two lines are fit from the glottal cycle peak to median values left and right. The lines are extended until 80% of ACFL is passed. The points of the slopes in the x-axis are the beginning and end of the open phase (see Fig 3(A)). Then open quotient is defined as the open phase divided by the period ($\text{OQ}=\frac{\text{t1}+\text{t2}}{\text{T0}}$), speed quotient as $\text{SQ}=\frac{\text{t1}}{\text{t2}}$, and the normalized amplitude quotient (NAQ) as $\text{NAQ}=\frac{\text{ACFL}}{\text{MFDR}\cdot \text{T0}}$.

**Table 2:** Frame-based glottal airflow measures estimated from the ambulatory neck-surface accelerometer signal using impedance-based inverse filtering.

Glottal airflow measures	Description	Units
**ACFL**	Peak-to-peak glottal airflow.	*mL*/*s*
**MFDR**	Negative peak of the first derivative of the glottal waveform.	*L*/*s*^2^
**Open Quotient (OQ)**	Ratio of the open time of the glottal vibratory cycle to the corresponding cycle period.	−
**Speed Quotient (SQ)**	Ratio of the opening time of the glottis to the closing time.	−
**H1-H2**	Difference between the magnitude of the first two harmonics.	dB
**Harmonic Richness Factor (HRF)**	Ratio of the sum of the amplitudes of the first 8 harmonics to the amplitude of the first harmonic.	dB
**Normalized Amplitude Quotient (NAQ)**	Ratio of ACFL to MFDR divided by the glottal period.	−
**logMFDR**	10*log*_10_|MFDR|^2^.	dB
**logACFL**	10*log*_10_|ACFL|^2^	dB
**MFDR’**	Ratio of estimated SPL (dB SPL) to logMFDR.	−
**ACFL’**	Ratio of estimated SPL (dB SPL) to logACFL.	−

We also included 4 additional measures derived from the time-domain measures:

- Logarithmic versions of ACFL and MFDR squared: 10log_10_|ACFL|^2^ (dB) and 10log_10_|MFDR|^2^ (dB).
- SPL normalized by ACFL (dB) and MFDR (dB): SPL/(10log_10_|ACFL|^2^) and SPL(/10log_10_|MFDR|^2^). Estimates for SPL are calculated using a linear regression equation: *y* = *mx* + *b*, where *m* and *b* are the coefficients from the subject obtained from accelerometer amplitude (*x*) and corresponding acoustic SPL (*y*). The calibration is done daily in the morning with a handheld microphone yielding the reference SPL [13], [35]. These ratios have shown to be significantly different between PVH and control subjects [4].

**Fig 4:**
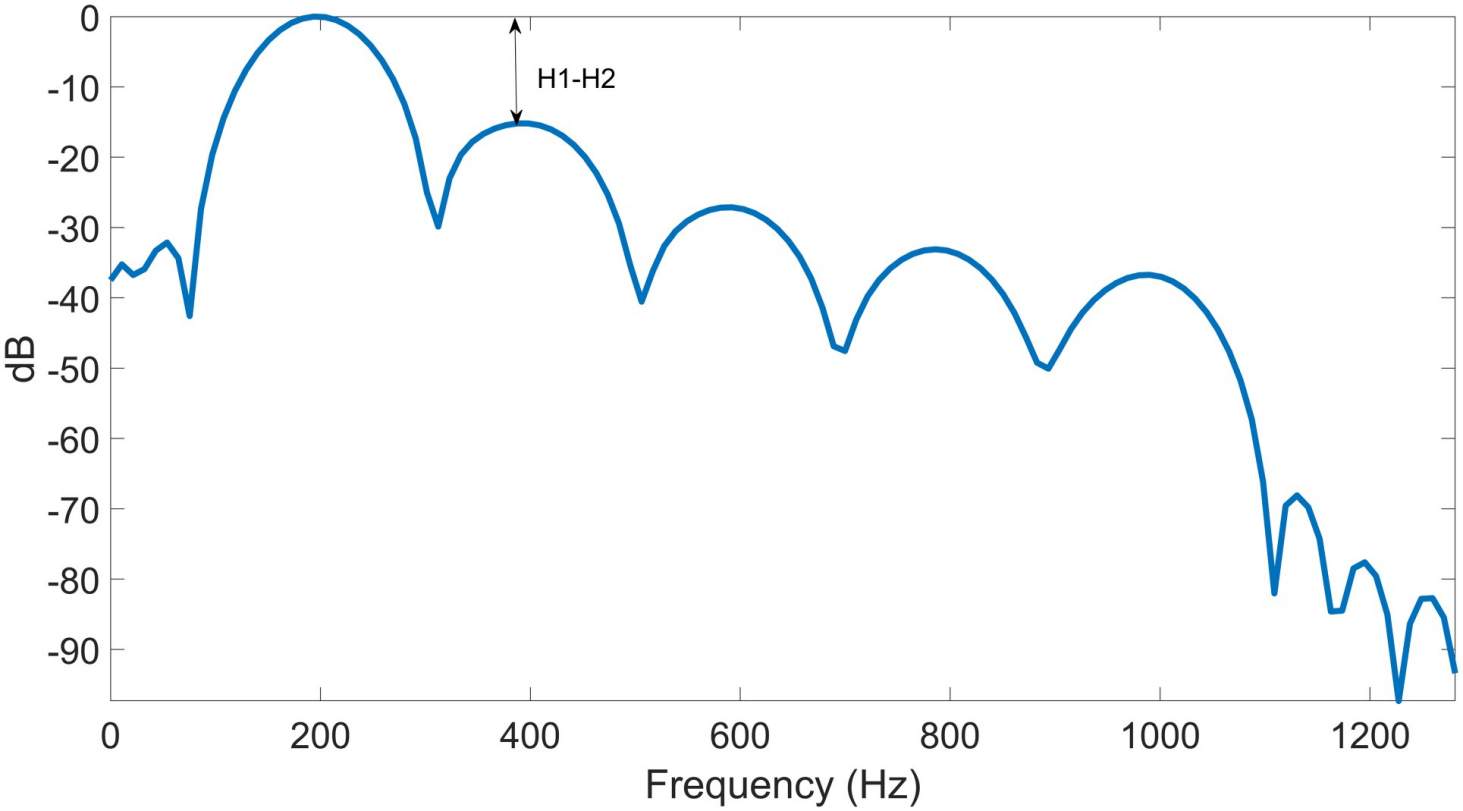
Spectrum of the frame in Fig 3(A).

Given that many of the glottal airflow features applied for vocal hyperfunction analysis are cycle-based [2], [4], [34] and multiple glottal cycles occur within each 50 ms frame, we computed average features across all glottal cycles in each frame. The idea was to provide a more consistent estimate of each measure, especially given the inherent fluctuations from continuous speech in the ambulatory signal. Fig 4 shows the spectrum of the estimated glottal airflow, from which spectral measures H1-H2 and harmonic richness factor (HRF) were computed. These measures have been correlated with voice quality [36], [37].

### Week-long univariate statistics for paired hypothesis testing

The purpose of the following series of tests is to find the most differentiating statistics between the PVH group and controls. Within-subject univariate statistics were calculated for each week-long time series data from each subject: mean, median, 5th percentile (trimmed minimum), 95th percentile (trimmed maximum), standard deviation, skewness, and kurtosis. These statistics were used for paired t-tests with 48 data points (number of subject pairs). Normality was tested with a Chi-square goodness-of-fit test, and each statistic was not significantly different from a normal distribution with *p* < 0.05. The false discovery rate is described by Eq (11), where *V* is the percentage of false positives (type I error) and *S* is the percentage of true positives. Since the false discovery rate is an expectation, we have *m* possible outcomes from the hypothesis tests.

$$\begin{array}{c} \text{False}\;\text{discovery}\;\text{rate}=E(\frac{V}{V+S}) \end{array}$$ (11)

If we have *H*_1_, *H*_2_,…*H*_*m*_ independent hypotheses, Benjamini-Hochberg (BH) [38] showed that regardless of how many null hypotheses are true and regardless of the distribution of the p-values, when the null hypothesis is false, we have the following property [39]:

$$\begin{array}{c} \text{False}\;\text{discovery}\;\text{rate}\leq \frac{U+V}{m}\alpha \leq \alpha \end{array}$$ (12)

where *U* is the proportion of true negatives. By setting *α* = 0.1, the procedure sorts the *m* p-values and defines a threshold *L*:

$$\begin{array}{c} L=\text{max}\{k:P_{k}\leq \frac{k}{m}\alpha \} \end{array}$$ (13)

We reject all hypotheses *H*_*k*_ for which *p*_*k*_ ≤ *p*_(*L*)_, the BH rejection threshold. This procedure will find those statistics with at most an *α* false discovery rate between PVH subjects and controls. It is important to remember that the false discovery rate is not the same as the type I error, but is the expected proportion of false positive features among the list of features that are significant according to the test. An example in reference [39] (page 687) uses a false discovery rate of 0.15, which is typical for analyses that are exploratory in nature [40]. In this case, we find the most differentiating statistics using this test, in contrast with a Bonferroni-corrected t-test, which yields a conservative comparison for which there is no statistically significant difference between any statistic.

### Supervised classification task

Following the same procedure as Ghassemi et al. [16], each subject’s weeklong ambulatory recording was subdivided into 5-minute windows (6000 frames, nonoverlapping). Only windows exhibiting voicing were only included in the classification task; voiced windows were defined as containing at least 0.5% voicing (30 voiced frames). We then calculated the following univariate statistics over the voiced frames within each window for each measure in Table I: mean, median, 5th percentile, 95th percentile, standard deviation, skewness, and kurtosis. Windows with less than 0.5% voicing were discarded due to data sparsity. Each window-based statistic was z-normalized (subtracting by the mean and dividing the result by the standard deviation) in two ways: a) by week, across voiced windows from all subjects (PVH and controls) and b) by day, across voiced windows within their respective days.

The full feature vector is composed of 154 features: 77 weekly and 77 daily z-score normalization the features derived from the 7 window-based univariate statistics for each of the 11 frame-based glottal airflow measures in Table 2. Since we only have a small amount of training data, we reduce feature dimensionality before training. As a first pass, forward feature selection (FFS) [41] is applied to the full feature matrix. The procedure is a greedy search algorithm that starts with an empty set *I* and iteratively selects a new feature *x* from the set of features not in *I* that minimizes a cost function *J* (a quadratic discriminant analysis classifier). The feature *x* is added to *I*, and the procedure is repeated until a threshold (10^−6^ in this case) of consecutive results is achieved. *E* is the quadratic discriminant analysis classification error using 5-fold cross-validation. The final reduced feature vector is composed of 55 features. It is worth mentioning that this subset is suboptimal since further reduction can be achieved through LASSO selection, which is applied later on. We use these features to build both logistic regression and support vector machine (SVM) supervised classifiers.

Logistic regression is a type of discriminative classifier that models the class-conditional probability as:

$$\begin{array}{c} P\left(y=1|x\right)=\frac{1}{1+e^{-x^{T}\beta }} \end{array}$$ (14)

where *x* ∈ *R*^*n*^ is the feature vector, *y* = 1 ∈ *R*^*l*^ is the class labeled as *y*_*i*_ = 1 (PVH) or *y*_*i*_ = 0 (control), and *β* is the vector of coefficient weights. In order to find the coefficients *β*, we maximize the following penalized log-likelihood using *N* data points of the training set with *p* feature vectors:

$$\begin{array}{c} \begin{array}{cc} & \max_{\beta \in R^{p+1}}\frac{1}{N}\sum_{i=1}^{N}\left\{y_{i}\text{log}p\left(x_{i}\right)+\left(1-y_{i}\right)\text{log}\left(1-p\left(x_{i}\right)\right)\right\}+\lambda {∥\beta ∥}_{1}, \end{array} \end{array}$$ (15)

where *x*_*i*_ is the data point for instance *i* and λ is the regularization parameter for the LASSO constraint. The *L*_1_ penalty reduces the number of features used in the model.

SVMs are commonly used machine learning tools for classification [42]. The weight vectors ***w*** ∈ *R*^*n*^ are optimized to create a linear *L*_1_ SVM classifier:

$$\begin{array}{c} \begin{array}{cc} & \min_{w}\;\sum_{i=1}^{N}{\left(\max\left(0,1-y_{i}w^{T}x_{i}\right)\right)}^{2}+C{∥w∥}_{1} \end{array} \end{array}$$ (16)

where *C* is a regularization parameter similar to λ for logistic regression. The goal is to create a sparse ***w*** that solves the *L*_2_-loss support vector classifier [43].

**Fig 5:**
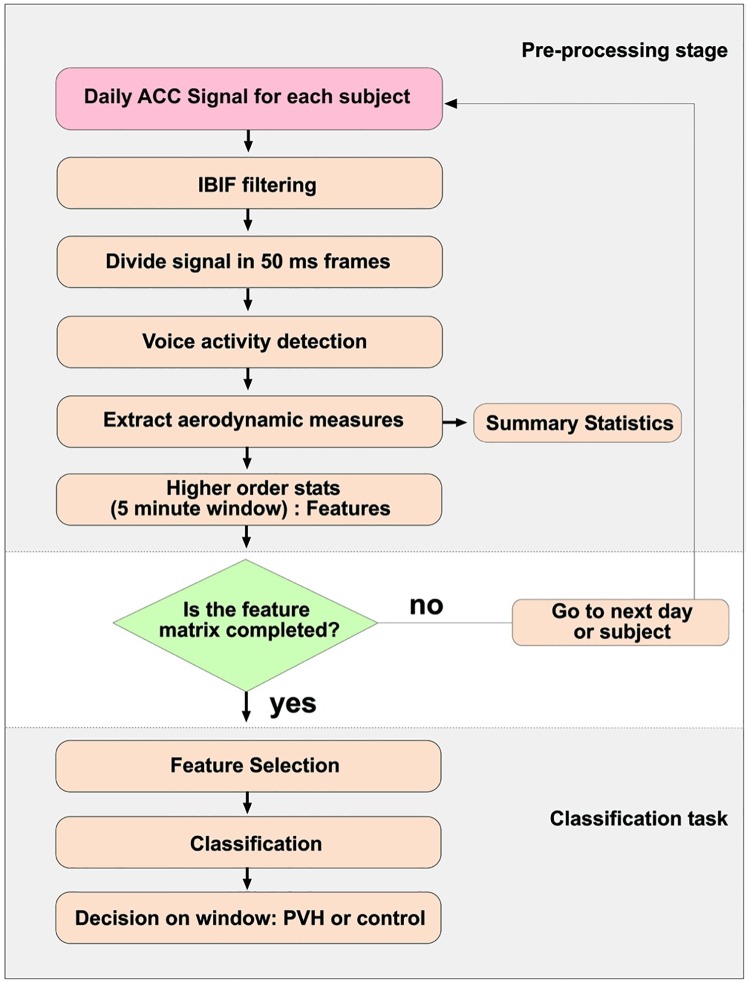
Flowchart. Feature extraction and classification process for 96 subjects.

Fig 5 shows a flowchart of the feature extraction and classification process. We first divided data using leave-one-out cross-validation to generate 48 datasets, each consisting of 47 training pairs and one test pair. All windows from the 47 training pairs (94 subjects total) were then subdivided using 5 cross-validation (1/5th validation and 4/5ths training in each fold). The validation sets are used to find the best set of parameters with respect to the area under the ROC curve (AUC) and these are selected for the model to be used in the test set. The following metrics are used to check the performance of the logistic model on the test pair: AUC, F-score, accuracy, sensitivity, specificity, positive predictive value (PPV), and negative predictive value (NPV). From this procedure, we test two scenarios: Classification with all the features after selection and using subsets of those features. The latter is done by sorting the absolute Beta values and running *L*_1_ logistic regression again by starting with all selected features. Then we took out the feature with lowest Beta value in magnitude and ran the classification again, and so on. The positive Beta weights are associated with subjects with PVH, whereas the negative weights are associated with control subjects.

## Results

### Week-long univariate statistics for paired hypothesis testing

Table 3 shows the first 11 features sorted from lowest to highest p-value from the paired t-tests *H*_1_…*H*_11_. The BH test rejects the first 8 null hypotheses *H*_1_…*H*_8_, i.e., they are significantly different at the 95% confidence level. Minimum and median ACFL were the most discriminative statistics, with medium effect sizes (Cohen’s *d* [44]) of 0.59 and 0.55, respectively. In general, statistics of the ACFL measure had the best differentiating power among all the week-long paired t-tests. In contrast, average values for estimated SPL for subjects from the same database were not significantly different between subjects with PVH and control subjects [15] [3]. This result suggests that high ACFL values are potentially good indicators of subjects with PVH, if the SPL distributions of both groups are statistically similar.

**Table 3:** Top 11 week-long summary statistics (from a total of 77) sorted by p-value from the 48 paired t-tests. Statistically significant differences (*) were found by applying the Benjamini-Hochberg method using a false discovery rate of 0.1.

Voice Use Summary Statistic	Patient Group	Matched-Control Group	p-value	Effect Size
**logACFL minimum**	38.5 ± 3.3	36.6 ± 2.9	0.0011*	0.59
**logACFL median**	49.2 ± 3.5	47.3 ± 3.7	0.0015*	0.55
**ACFL minimum**	90.8 ± 40.6	71.6 ± 23.5	0.0016*	0.58
**logACFL mean**	48.7 ± 3.5	46.9 ± 3.5	0.0025*	0.52
**ACFL median**	315 ± 140	251 ± 99.0	0.0030*	0.53
**H1-H2 kurtosis**	10.9 ± 4.30	8.8 ± 2.6	0.0061*	0.59
**logACFL kurtosis**	3.17 ± 0.60	2.93 ± 0.4	0.0076*	0.50
**ACFL mean**	359 ± 163	296 ± 117	0.0091*	0.45
**H1-H2 minimum**	2.39 ± 4.20	0.38 ± 4.4	0.0120	0.48
**HRF kurtosis**	11.6 ± 4.7	10.0 ± 2.9	0.0230	0.42
**MFDR median**	365.4 ± 171.8	310.6 ± 127.7	0.0270	0.37

### Supervised classification task

**Fig 6:**
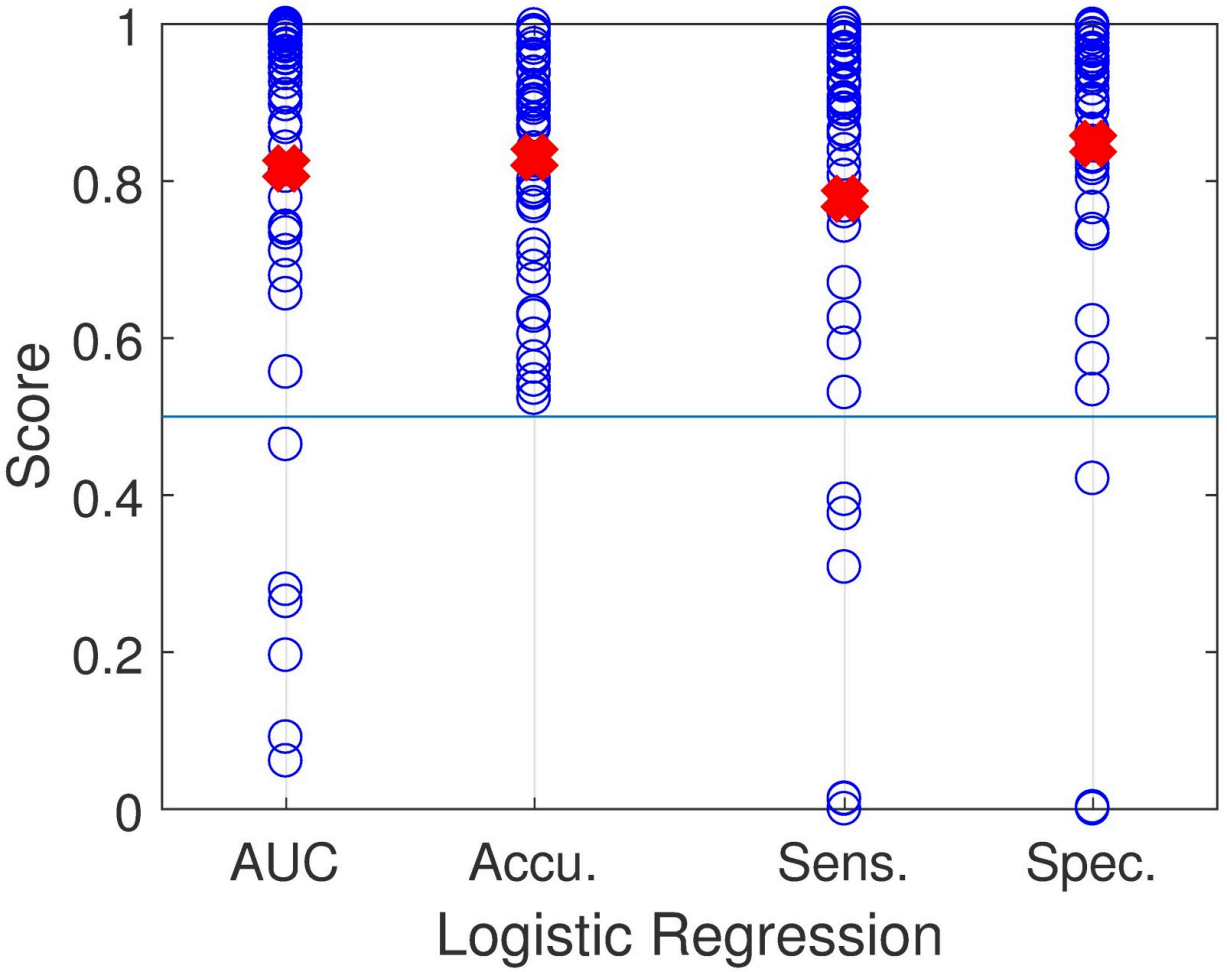
Performance results across subject pairs with L1-logistic regression. Area Under the ROC Curve (AUC), Accuracy, Sensitivity, Specificity. The red crosses indicates the average value for each performance metric.

Table 4 shows a summary of the classification results for both implemented classifiers using the multiple performance metrics. Fig 6 displays performance of the *L*_1_ logistic regression classifier for each of the 48 pairs for a subset of the performance metrics. There is a large spread of AUC scores across the subjects with an average of 0.82. AUC scores less than 0.5 indicate that the model places weight on positive examples versus negative ones and vice versa. The large AUC variance, including values less than 0.5, could be explained from the labels; e.g., subjects with PVH do not always exhibit vocal behavior typical for the pathology, whereas control subjects might exhibit some vocal behavior that differs substantially from healthy vocal behavior.

**Table 4:** Classification performance of L1 logistic regression (L1-LR) and support vector machine (SVM) approaches for 96 subjects using IBIF features. Mean (standard deviation) is reported for the performance metrics. Previous results using 51 pairs [3] and 20 pairs [16] are also shown. It is worth noting that the distribution of metrics such as AUC, across all models, may be non-normal and may benefit from other summary statistics such as median (IQR).

Method	AUC	Accuracy	F-score	Sensitivity	Specificity	PPV	NPV	Threshold
**L1-LR (IBIF)**	0.82 (0.25)	0.83 (0.14)	0.77 (0.27)	0.78 (0.29)	0.85 (0.22)	0.81 (0.21)	0.82 (0.18)	0.54 (0.25)
**SVM (IBIF)**	0.82 (0.26)	0.84 (0.14)	0.78 (0.27)	0.79 (0.28)	0.84 (0.24)	0.83 (0.22)	0.82 (0.21)	0.02 (0.67)
**Mehta et al**. [3]	0.74 (0.27)	-	0.77 (0.20)	0.74 (0.30)	0.77 (0.29)	-	-	-
**Ghassemi et al**. [16]	0.71 (-)	0.66 (-)	0.63 (-)	0.50 (-)	0.81 (-)	0.72 (-)	0.62 (-)	-

**Fig 7:**
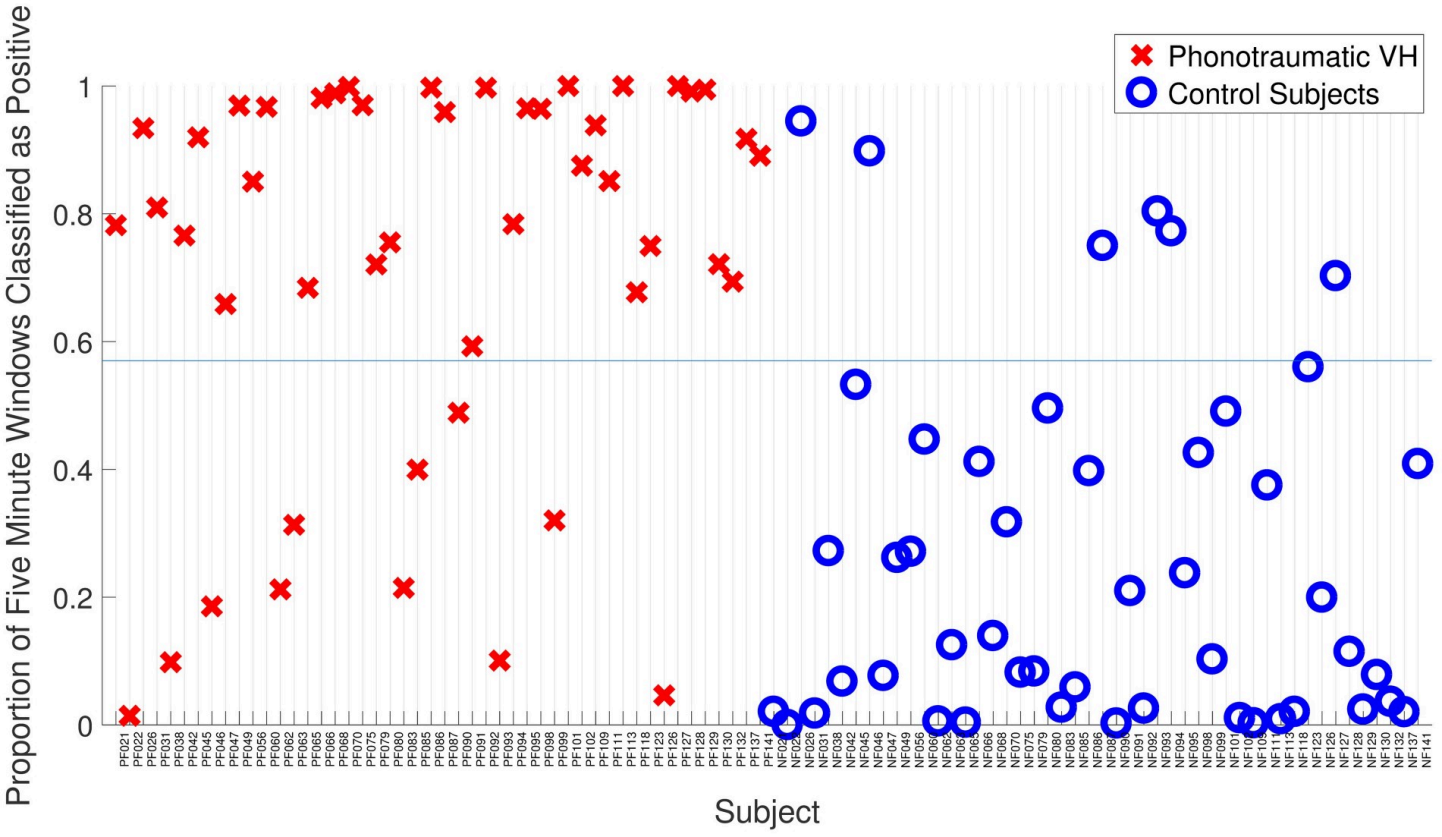
Classification results from L1-logistic regression. The threshold (blue line) at 0.57 classifies correctly 79 from 96 subjects (82.3%).

Logistic regression and SVM have similar good results on all performance metrics. Since L1 regularization was used in both cases, it could be that the removal of redundant features in every training case helped the performance. The mean (standard deviation) of the performance metrics for both classifiers improved when compared with previous results on 51 matched-paired subjects: 0.74 (0.27) for AUC, 0.77 (0.20) for F-score, 0.74 (0.30) for sensitivity, and 0.77 (0.29) for specificity [3]. Fig 7 shows the proportion of labels classified as positive (VH) for all subjects. 79 subjects from 96 were classified correctly by using a threshold of 0.57. This corresponds to 82.3% of accuracy.

**Fig 8:**
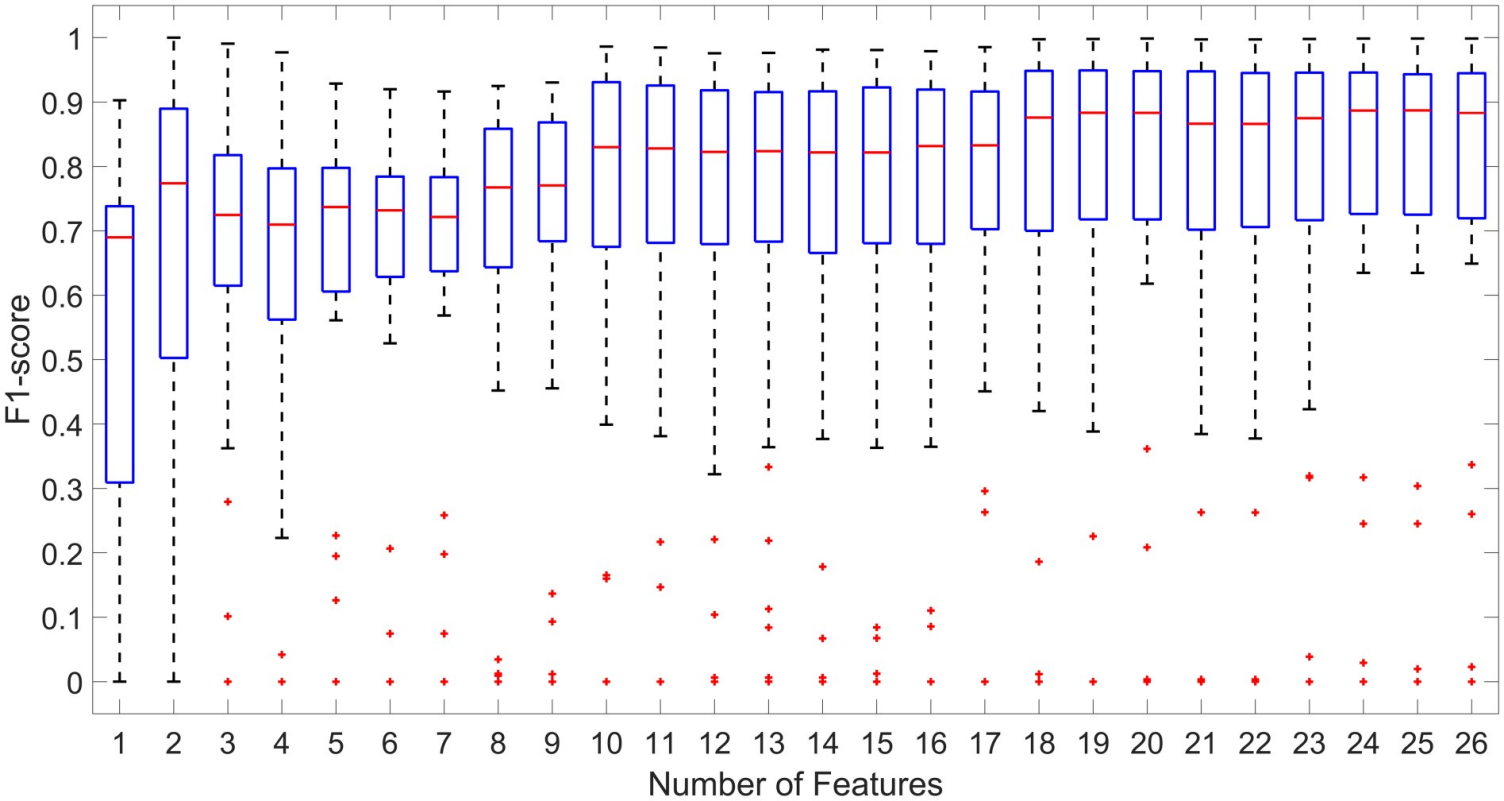
F-score distributions from Table 5. From all 26 features (rightmost box plot) to only one feature (H1-H2 95th%, leftmost box plot).

Feature selection is important for identifying the most relevant features that can help to further understand the underlying process, as well as reducing the complexity for future biofeedback applications. Table 5 shows the total number of features (26) that were present in all 48 models after using LASSO with the resulting 55 features after FFS. Table 6 shows the results for all 26 models and the subset of features by sorting beta values. The mean F-score is stable in the 0.7 region until the number of features is 9. After that, the performance degrades moderately, where the AUC is 0.68 and the accuracy is 0.71 with only 7 features. Fig 8 shows boxplots of the same models versus F-score, where we can see the same trend: classification performance is more or less similar if we left in 9 features or more in the classifier.

**Table 5:** Association count of Beta (weight) variables that were included in all 48 models. These 26 features were present in each logistic regresion model.

Associated Feature	Phonotraumatic	Control	Beta Weight Mean	Standard Deviation
**H1-H2 95th% (Daily Normalized)**	48	0	2.50	0.16
**NAQ mean**	48	0	1.42	0.11
**HRF skewness**	48	0	1.38	0.09
**logACFL standard deviation**	48	0	1.30	0.10
**HRF 5th% (daily normalized)**	48	0	1.21	0.15
**logACFL skewness (daily normalized)**	48	0	1.17	0.05
**SQ 5th%**	48	0	1.16	0.05
**SQ standard deviation**	48	0	1.12	0.06
**MFDR’ 95th%**	48	0	1.01	0.13
**OQ 5th%**	48	0	0.94	0.12
**H1-H2 standard deviation (daily normalized)**	48	0	0.71	0.09
**HRF dtandard deviation (daily normalized)**	48	0	0.43	0.06
**logMFDR 5th% (daily normalized)**	48	0	0.32	0.06
**ACFL’ standard deviation (daily normalized)**	48	0	0.18	0.02
**SQ skewness (daily normalized)**	0	48	-0.12	0.03
**SQ standard deviation (daily normalized)**	0	48	-0.21	0.02
**OQ 5th% (daily normalized)**	0	48	-0.27	0.04
**SQ 5th% (daily normalized)**	0	48	-0.41	0.02
**NAQ mean (daily normalized)**	0	48	-0.47	0.05
**HRF skewness (daily normalized)**	0	48	-0.89	0.06
**logACFL standard deviation (daily normalized)**	0	48	-0.97	0.07
**OQ mean**	0	48	-1.00	0.13
**H1-H2 dtandard deviation**	0	48	-1.28	0.13
**logACFL skewness**	0	48	-1.58	0.07
**HRF 5th%**	0	48	-1.86	0.29
**H1-H2 95th%**	0	48	-4.47	0.31

**Table 6:** Mean and (standard deviation) performance metrics from L1-logistic regression for different group of features from Table 5, starting with the whole set of 26 features. Iteratively, the following group is obtained by taking out the feature with the smallest absolute Beta value.

Added feature	Number	AUC	F-score	Accuracy	Sensitivity	Specificity	PPV	NPV
Daily norm. Log ACFL Skew	26	0.82 (0.25)	0.76 (0.30)	0.84 (0.14)	0.77 (0.32)	0.87 (0.19)	0.79 (0.27)	0.83 (0.19)
Daily norm. ACFL’ stand. dev.	25	0.82 (0.25)	0.76 (0.30)	0.83 (0.14)	0.77 (0.32)	0.87 (0.19)	0.79 (0.27)	0.83 (0.19)
Daily norm. SQ stand. dev.	24	0.82 (0.25)	0.76 (0.30)	0.84 (0.14)	0.77 (0.32)	0.87 (0.19)	0.79 (0.27)	0.83 (0.19)
Daily norm. OQ 5th%	23	0.82 (0.25)	0.77 (0.28)	0.83 (0.14)	0.77 (0.30)	0.86 (0.19)	0.80 (0.24)	0.83 (0.19)
Daily norm Log MFDR 5th%	22	0.82 (0.25)	0.77 (0.28)	0.83 (0.15)	0.78 (0.30)	0.85 (0.23)	0.81 (0.25)	0.82 (0.22)
Daily norm. SQ 5th%	21	0.82 (0.25)	0.77 (0.28)	0.83 (0.15)	0.78 (0.30)	0.85 (0.22)	0.81 (0.25)	0.81 (0.22)
Daily norm. HRF stand. dev.	20	0.82 (0.27)	0.78 (0.28)	0.83 (0.15)	0.79 (0.30)	0.85 (0.23)	0.81 (0.25)	0.82 (0.22)
Daily norm. NAQ mean	19	0.82 (0.27)	0.78 (0.28)	0.84 (0.15)	0.79 (0.30)	0.85 (0.22)	0.79 (0.27)	0.82 (0.22)
Daily norm. H1-H2 stand. dev.	18	0.82 (0.26)	0.77 (0.28)	0.83 (0.15)	0.78 (0.30)	0.85 (0.22)	0.80 (0.25)	0.81 (0.22)
Daily norm. HRF skew	17	0.79 (0.24)	0.74 (0.28)	0.80 (0.14)	0.75 (0.30)	0.81 (0.23)	0.76 (0.26)	0.78 (0.21)
OQ 5th%	16	0.77 (0.24)	0.71 (0.30)	0.79 (0.15)	0.73 (0.33)	0.80 (0.26)	0.74 (0.26)	0.77 (0.21)
Daily norm. Log ACFL stand. dev.	15	0.77 (0.24)	0.71 (0.30)	0.79 (0.14)	0.73 (0.32)	0.80 (0.25)	0.77 (0.24)	0.77 (0.21)
OQ mean	14	0.78 (0.24)	0.72 (0.29)	0.79 (0.14)	0.73 (0.32)	0.80 (0.26)	0.77 (0.24)	0.78 (0.21)
MFDR’ 95th%	13	0.78 (0.24)	0.71 (0.29)	0.79 (0.14)	0.72 (0.32)	0.81 (0.25)	0.78 (0.21)	0.77 (0.21)
SQ stand. dev.	12	0.78 (0.24)	0.71 (0.30)	0.79 (0.14)	0.72 (0.33)	0.82 (0.25)	0.77 (0.24)	0.77 (0.21)
SQ 5th%	11	0.78 (0.24)	0.73 (0.27)	0.79 (0.14)	0.75 (0.30)	0.79 (0.26)	0.76 (0.24)	0.78 (0.21)
Daily norm. Log ACFL skew	10	0.78 (0.25)	0.73 (0.28)	0.79 (0.15)	0.75 (0.31)	0.79 (0.27)	0.76 (0.24)	0.78 (0.21)
Daily norm. HRF 5th%	9	0.75 (0.23)	0.71 (0.25)	0.75 (0.13)	0.74 (0.28)	0.72 (0.30)	0.73 (0.20)	0.75 (0.19)
H1-H2 stand. dev.	8	0.74 (0.22)	0.69 (0.26)	0.75 (0.13)	0.72 (0.29)	0.72 (0.29)	0.73 (0.20)	0.73 (0.19)
Log ACFL stand. dev.	7	0.68 (0.22)	0.62 (0.29)	0.71 (0.12)	0.66 (0.33)	0.69 (0.28)	0.63 (0.26)	0.66 (0.23)
HRF skew	6	0.69 (0.22)	0.63 (0.29)	0.71 (0.12)	0.67 (0.32)	0.68 (0.29)	0.63 (0.26)	0.67 (0.23)
NAQ mean	5	0.68 (0.23)	0.63 (0.29)	0.71 (0.12)	0.68 (0.34)	0.68 (0.29)	0.63 (0.26)	0.68 (0.24)
Log ACFL skew	4	0.66 (0.24)	0.63 (0.27)	0.70 (0.13)	0.67 (0.32)	0.67 (0.33)	0.64 (0.25)	0.66 (0.23)
HRF 5th%	3	0.63 (0.30)	0.64 (0.29)	0.71 (0.15)	0.69 (0.34)	0.67 (0.36)	0.66 (0.37)	0.74 (0.22)
Daily norm. H1-H2 95th%	2	0.63 (0.33)	0.64 (0.33)	0.74 (0.16)	0.67 (0.38)	0.76 (0.34)	0.69 (0.32)	0.70 (0.29)
H1-H2 95th%	1	0.58 (0.22)	0.53 (0.30)	0.65 (0.10)	0.58 (0.37)	0.64 (0.35)	0.58 (0.26)	0.64 (0.16)

**Fig 9:**
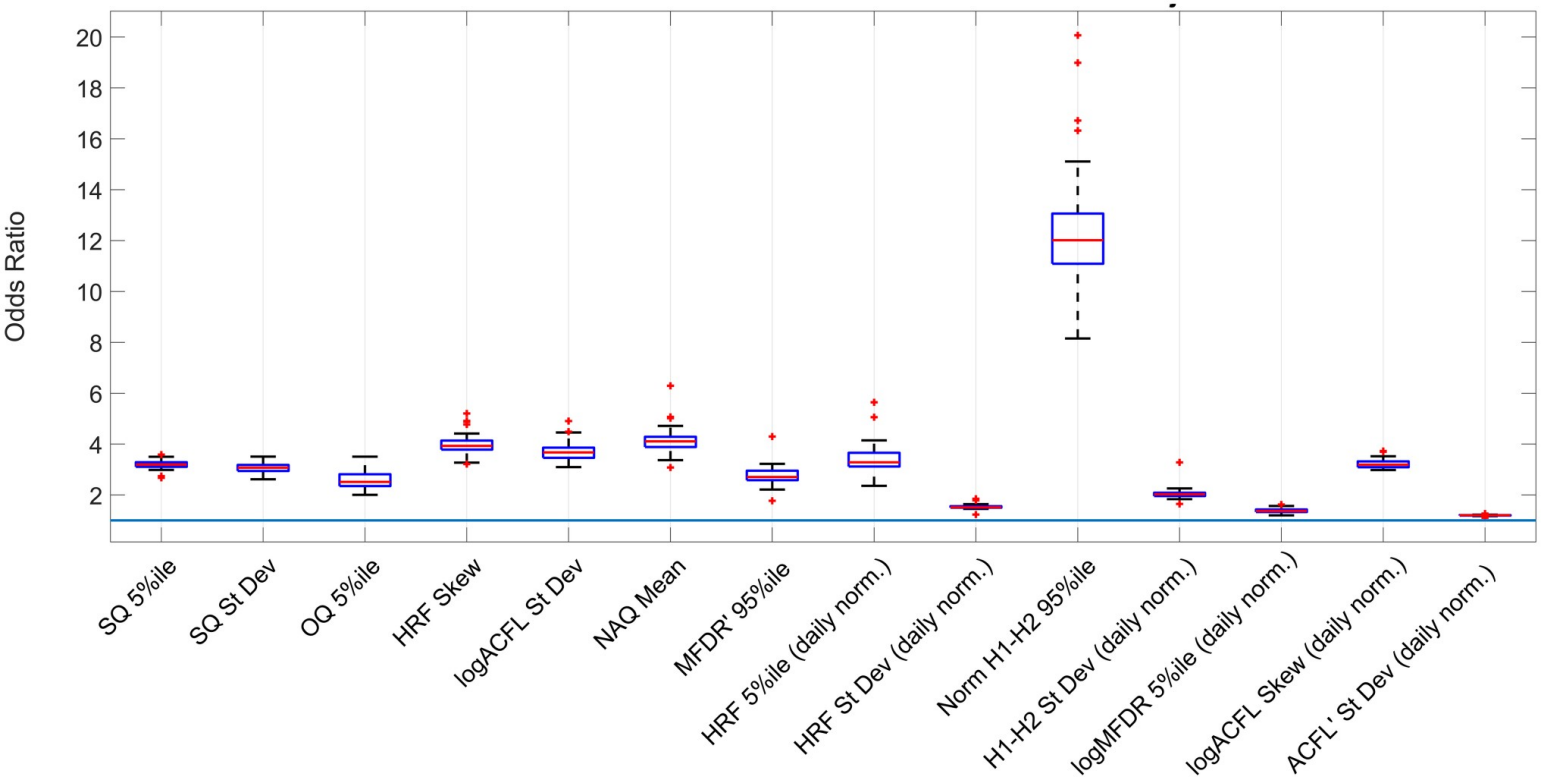
Odds ratio association with phonotraumatic subjects.

Fig 9 shows the association counts of features with PVH subjects as odds ratios. Odds ratios represent the association with a one-unit increase in the features. These features represent a combination of time and frequency-domain features that were consistently present in all 48 logistic regression models with *p* < 0.05 [3]. The 95th percentile of H1-H2 (daily normalized) had a large association with PVH labels, which is a voice measure correlated with voice quality [36]. However, the large confidence interval for this feature represents low level of precision of the odds ratio. The 95th percentile ratio of SPL and MFDR (MFDR’ 95%ile in Fig 9) has a moderate association compared to the rest of the features with a small confidence interval, representing a higher precision on the odds ratio.

The current study sought to determine whether optimized IBIF-based estimates of glottal airflow measures extracted from ambulatory voice (accelerometer-based) recordings can be used to differentiate between normal vocal function and pathophysiological mechanisms associated with PVH. Results showed that this approach can be quite successful in classifying subjects as being normal or having PVH. Within-subject univariate analyses identified eight aerodynamic features that were statistically different between the patient and matched control groups. ACFL was the most significant measure with medium effect sizes exhibited. These findings are in agreement with previous laboratory studies that used measures extracted from the inverse filtered oral airflow [2], [4] and with computer modeling that suggests that increases in ACFL may reflect the type of increased compensatory effort (i.e., increased vocal hyperfunction) that is necessary for PVH patients to maintain adequate phonation in the presence of vocal fold fold trauma/lesions [9], [10]. Such increases in vocal effort are believed to reflect the “vicious cycle” of progressive concomitant increases in PVH and vocal fold trauma that contribute to perpetuating these disorders.

From Table 4, use of the IBIF-based glottal airflow measures in the supervised classification task produced results that outperformed previous reports that used acoustic-based features extracted from ambulatory recordings of the acceleration signal to differentiate between subjects with PVH and normal controls [16], [3]. The improvement in performance using IBIF-based features, in combination with the capability of such features to provide better insights into pathophysiological mechanisms, supports the potential that this approach has to improve the clinical assessment of hyperfunctional voice disorders. Future research could explore the performance of IBIF-based features with other pathologies, such as unilateral vocal fold paralysis [11].

There are several limitations to the current study which may serve to constrain any addition improvement in classification performance. First, even though the use of univariate statistics over 5-minute windows showed good performance, such an approach could smooth out fast variations in some features that may provide important information related to pathophysiology. Moreover, discarding silence periods from the analysis windows might be eliminating information that could further differentiate normal and pathological vocal function by indicating relative differences in non-vocal (non-phonatory) recovery times.

In addition, determination of the IBIF Q parameters is based on accurate estimates of the glottal volume velocity waveform obtained by inverse filtering the oral airflow recorded in the laboratory during sustained vowel production. However, the process of inverse filtering to estimate the glottal flow is still a topic of research and any method will have a degree of error (see [7], [8] for general discussions). The inverse filtering process is particularly challenging when applied to pathological female voices, as was done in this study. The process was made even more demanding by that fact that many subjects in this study were singers who regularly reached very high pitches (above 400 Hz) daily during practice that tend to cause the inverse filtering and IBIF methods to fail. In addition, every feature has an associated uncertainty from the accelerometer measurements, and the task becomes difficult when we combine multiple estimated features (e.g., for the SPL-normalized measures of ACFL’ and MFDR’) since those errors may propagate and increase the total uncertainty in an ambulatory setting.

Finally, the task of differentiating normal and pathological subjects was made more difficult because the patients with PVH in this study were classified as having only mild-to-moderate voice disorders. We know from clinical experience that such patients can display periods of seemingly normal vocal function, and, conversely, normal speakers can display transient episodes of VH that do not develop into chronic conditions. Future studies could attempt to address these issues by developing estimates of uncertainty for the extracted IBIF parameters and using other analysis methods such as unsupervised learning to better pinpoint specific segments of abnormal vocal function, as has been initially demonstrated in [45]. In addition, efforts to incorporate aerodynamic features in the framework of ambulatory biofeedback to improve voice therapy are currently underway [46].

## Conclusion

An ambulatory approach that correctly identifies the instance, duration, and type of incorrect vocal behaviors during daily activities has the capability to provide transformative advancements for the assessment, monitoring, and treatment of vocal hyperfunction. In this study, we further develop prior ambulatory efforts, by improving the ability to discriminate pathological voices from healthy ones. Using an impedance-based inverse filtering scheme to estimate the unsteady glottal airflow component from a neck-surface accelerometer and a smartphone platform, we obtain and quantify, for the first time in an ambulatory assessment and a comprehensive framework, aerodynamic features that have been shown to be physiologically relevant for vocal hyperfunction in recent laboratory settings and computational studies. Prior efforts to obtain aerodynamic features from neck surface acceleration were limited to sustained vowels [18] and simple proof of concept examples [3]. The result of our comprehensive quantitative analysis show that these ambulatory glottal airflow measures can be successfully used to differentiate between normal vocal function and pathophysiological mechanisms associated with phonotraumatic vocal hyperunction, and outperform state-of-the-art reports using sound pressure level, fundamental frequency, and related vocal doses. Due to its physiological relevance, the proposed aerodynamic ambulatory approach has already potential to improve the clinical assessment of hyperfunctional voice disorders, including the evaluation of treatment outcomes. Thus, future efforts will be focused on further relating ambulatory aerodynamic features to vocal therapy and real-time biofeedback.

## References

[1] Roy N, Merrill RM, Gray SD, Smith EM. Voice Disorders in the General Population: Prevalence, Risk Factors, and Occupational Impact. The Laryngoscope. 2005;115(11):1988–1995. 10.1097/01.mlg.0000179174.32345.41 16319611

[2] Hillman RE, Holmberg EB, Perkell JS, Walsh M, Vaughan C. Objective Assessment of Vocal Hyperfunction: An Experimental Framework and Initial Results. J Speech Hear Res. 1989;32:373–392. 10.1044/jshr.3202.373 2739390

[3] Mehta DD, Van Stan JH, Zañartu M, Ghassemi M, Guttag JV, Espinoza VM, et al Using ambulatory voice monitoring to investigate common voice disorders: research update. Front Bioeng Biotechnol 3:155 2015;. 10.3389/fbioe.2015.00155 26528472 PMC4607864

[4] Espinoza VM, Zañartu M, Van Stan JH, Mehta DD, Hillman RE. Glottal Aerodynamic Measures in Women With Phonotraumatic and Nonphonotraumatic Vocal Hyperfunction. Journal of Speech, Language, and Hearing Research. 2017;60(8):2159–2169. 10.1044/2017_JSLHR-S-16-0337 28785762 PMC5829799

[5] Rothenberg M. A new inverse filtering technique for deriving the glottal air flow waveform during voicing. The Journal of the Acoustical Society of America. 1973;53(6):1632–1645. 10.1121/1.1913513 4719255

[6] Perkell JS, Hillman RE, Holmberg EB. Group differences in measures of voice production and revised values of maximum airflow declination rate. The Journal of the Acoustical Society of America. 1994;96(2):695–698. 10.1121/1.410307 7930069

[7] Alku P. Glottal inverse filtering analysis of human voice production: A review of estimation and parameterization methods of the glottal excitation and their applications. SADHANA—Academy Proceedings in Engineering Sciences. 2011;36:623–650.

[8] Drugman T, Alku P, Alwan A, Yegnanarayana Y. Glottal source processing: From analysis to applications. Computer Speech & Language. 2014;28:1117–1138. 10.1016/j.csl.2014.03.003

[9] Galindo GE, Peterson SD, Erath BD, Castro C, Hillman RE, Zañartu M. Modeling the pathophysiology of phonotraumatic vocal hyperfunction with a triangular glottal model of the vocal folds. Journal of Speech, Language, and Hearing Research. 2017;60(9):2452–2471. 10.1044/2017_JSLHR-S-16-0412 28837719 PMC5831616

[10] Zañartu M, Galindo G, Erath BD, Peterson SD, Wodicka GR, Hillman RE. Modeling the effects of a posterior glottal opening on vocal fold dynamics with implications for vocal hyperfunction. J Acoust Soc Am. 2014;136:3262–3271. 10.1121/1.4901714 25480072 PMC4257958

[11] Pinho CMR, Jesus LMT, Barney A. Aerodynamic measures of speech in unilateral vocal fold paralysis (UVFP) patients. Logopedics Phoniatrics Vocology. 2013;38(1):19–34. 10.3109/14015439.2012.696138 22741532

[12] Zraick RI, Smith-Olinde L, Shotts LL. Adult Normative Data for the KayPENTAX Phonatory Aerodynamic System Model 6600. Journal of Voice. 2012;26(2):164–176. 10.1016/j.jvoice.2011.01.006 21600731

[13] Mehta DD, Zañartu M, Feng SW, Cheyne HA, Hillman RE. Mobile Voice Health Monitoring Using a Wearable Accelerometer Sensor and a Smartphone Platform. Biomedical Engineering, IEEE Transactions on. 2012;59(11):3090–3096. 10.1109/TBME.2012.2207896 PMC3539821 22875236

[14] Titze IR, Svec JG, Popolo PS. Vocal Dose Measures: Quantifying Accumulated Vibration Exposure in Vocal Fold Tissues. J Speech Lang Hear Res. 2003;46:919–932. 10.1044/1092-4388(2003/072) 12959470 PMC3158591

[15] Van Stan JH, Mehta DD, Zeitels SM, Burns JA, Barbu AM, Hillman RE. Average Ambulatory Measures of Sound Pressure Level, Fundamental Frequency, and Vocal Dose Do Not Differ Between Adult Females With Phonotraumatic Lesions and Matched Control Subjects. Annal Otolog Rhinol Laryngol. 2015;124(11):864–874. 10.1177/0003489415589363 PMC4605885 26024911

[16] Ghassemi M, Van Stan JH, Mehta DD, Zañartu M, Cheyne HA, Hillman RE, et al Learning to Detect Vocal Hyperfunction From Ambulatory Neck-Surface Acceleration Features: Initial Results for Vocal Fold Nodules. Biomedical Engineering, IEEE Transactions on. 2014;61(6):1668–1675. 10.1109/TBME.2013.2297372 PMC4077201 24845276

[17] Muhammad G, Alhamid MF, Alsulaiman M, Gupta B. Edge Computing with Cloud for Voice Disorder Assessment and Treatment. IEEE Communications Magazine. 2018;56(4):60–65. 10.1109/MCOM.2018.1700790

[18] Zañartu M, Ho JC, Mehta DD, Hillman RE, Wodicka GR. Subglottal Impedance-Based Inverse Filtering of Voiced Sounds Using Neck Surface Acceleration. Audio, Speech, and Language Processing, IEEE Transactions on. 2013;21(9):1929–1939. 10.1109/TASL.2013.2263138 PMC4229092 25400531

[19] Harper P, Kraman SS, Pasterkamp H, Wodicka GR. An Acoustic Model of the Respiratory Tract. J Appl Physiol. 2001;77:554–566. 10.1109/10.918593 11341528

[20] Ho JC, Zañartu M, Wodicka GR. An Anatomically Based, Time-Domain Acoustic Model of the Subglottal System for Speech Production. J Acoust Soc Am. 2011;129(3):1531–1547. 10.1121/1.3543971 21428517

[21] Proakis JG, Manolakis DG. Digital Signal Processing: Principles, Algorithms and Applications. 4th ed Pearson Education Inc; 2007.

[22] Ishizaka K, French JC, Flanagan JL. Direct Determination of Vocal Tract Wall Impedance. IEEE Transaction on Acoustics, Speech and Signal Processing. 1975;23:370–373. 10.1109/TASSP.1975.1162701

[23] Kennedy J, Eberhart R. Particle swarm optimization. In: Neural Networks, 1995. Proceedings., IEEE International Conference on. vol. 4; 1995. p. 1942–1948 vol.4.

[24] Rabiner LR. Digital Processing of Speech Signals. Prentice Hall; 1978.

[25] Zañartu M. Acoustic Coupling in Phonation and its Effect on Inverse Filtering of Oral Airflow and Neck Surface Acceleration. Purdue University West Lafayette, IN; 2010.

[26] Mehta DD, Hillman RE. Voice Assessment: Updates on Perceptual, Acoustic, Aerodynamic, and Endoscopic Imaging Methods. Curr Opin Otolaryngol Head Neck Surg. 2008;16:211–215. 10.1097/MOO.0b013e3282fe96ce 18475073 PMC3775647

[27] Kunduk M, Mcwhorter A. True vocal fold nodules: The role of differential diagnosis. 2009;17:449–52. 10.1097/MOO.0b013e3283328b6d 19779347

[28] Alisa Z, Danielle B, M KS, Thomas M, Lucian S. Gender and age in benign vocal fold lesions. The Laryngoscope;125(1):191–196. 25216037 10.1002/lary.24911

[29] Perkell JS, Holmberg EB, Hillman RE. A system for signal processing and data extraction from aerodynamic, acoustic, and electroglottographic signals in the study of voice production. The Journal of the Acoustical Society of America. 1991;89(4):1777–1781. 10.1121/1.401011 2045586

[30] Mehta DD, Zañartu M, Van Stan JH, Feng SW, Cheyne H, Hillman RE. Smartphone-based detection of voice disorders by long-term monitoring of neck acceleration features. IEEE 10th Annual Wearable and Implantable Body Sensor Networks Conference. 2013. Cambridge, USA.

[31] Espinoza VM, Zañartu M, Van Stan JH, Mehta DD, Hillman RE. Uncertainty of glottal airflow estimation during continuous speech using impedance-based inverse filtering of the neck-surface acceleration signal. The Journal of the Acoustical Society of America. 2017;141(5):3579–3579. 173rd Meeting of The ASA. Boston, USA. 10.1121/1.4987622

[32] Alku P, Pohjalainen J, Vainio M, Laukkanen A, Story B. Formant frequency estimation of high-pitched vowels using weighted linear prediction. The Journal of the Acoustical Society of America. 2013;134(2):1295–1313. 10.1121/1.4812756 23927127

[33] Alku P, Horáĉek J, Airas M, Griffond-Boitier F, Laukkanen AM. Performance of Glottal Inverse Filtering as Tested by Aeroelastic Modelling of Phonation and FE Modelling of Vocal Tract. Acta Acustica united with Acustica. 2006;92(5):717–724.

[34] Holmberg EB, Hillman RE, Perkell JS. Glottal Air-Flow and Transglottal Air-Pressure Measurements for Male and Female Speakers in Soft, Normal, and Loud Voice. J Acoust Soc Am. 1988;84:511–529. 10.1121/1.396829 3170944

[35] Švec JG, Titze IR, Popolo PS. Estimation of sound pressure levels of voiced speech from skin vibration of the neck. The Journal of the Acoustical Society of America. 2005;117(3):1386–1394. 10.1121/1.1850074 15807026

[36] Klatt DH, Klatt LC. Analysis, Synthesis and Perception of Voice Quality Variations Among Male and Female Talkers. J Acoust Soc Am. 1990;87(2):820–856. 10.1121/1.398894 2137837

[37] Kreiman J, Shue YL, Chen G, Iseli M, Gerratt BR, Neubauer J, et al Variability in the relationships among voice quality, harmonic amplitudes, open quotient, and glottal area waveform shape in sustained phonation. The Journal of the Acoustical Society of America. 2012;132(4):2625–2632. 10.1121/1.4747007 23039455 PMC3477193

[38] Benjamini Y, Hochberg Y. Controlling the False Discovery Rate: A Practical and Powerful Approach to Multiple Testing. Journal of the Royal Statistical Society Series B (Methodological). 1995;57(1):289–300. 10.1111/j.2517-6161.1995.tb02031.x

[39] Hastie T, Tibshirani R, Friedman J. The Elements of Statistical Learning: Data Mining, Inference, and Prediction. Springer; 2009.

[40] Rieger KE, Hong WJ, Tusher VG, Tang J, Tibshirani R, Chu G. Toxicity from radiation therapy associated with abnormal transcriptional responses to DNA damage. Proceedings of the National Academy of Sciences of the United States of America. 2004;101(17):6635–6640. 10.1073/pnas.0307761101 15096622 PMC404097

[41] Kohavi R, John GH. Wrappers for feature subset selection. Artificial Intelligence. 1997;97(1):273–324. 10.1016/S0004-3702(97)00043-X

[42] Boser BE, Guyon IM, Vapnik VN. A Training Algorithm for Optimal Margin Classifiers. In: Proceedings of the Fifth Annual Workshop on Computational Learning Theory. COLT’92; 1992. p. 144–152.

[43] Fan RE, Chang KW, Hsieh CJ, Wang XR, Lin CJ. LIBLINEAR: A Library for Large Linear Classification. J Mach Learn Res. 2008;9:1871–1874.

[44] Cohen J. Statistical Power Analysis for the Behavioral Sciences. 2nd ed Hillsdale: Lawrence Erlbaum; 1988.

[45] Ghassemi M, Syed Z, Mehta D, Van Stan J, Hillman R, Guttag J. Uncovering voice misuse using symbolic mismatch. Machine Learning for Healthcare Conference. 2016;56:239–252. PMC8693775 34950284

[46] Llico AF, Zañartu M, González AJ, Wodicka GR, Mehta DD, Stan JHV, et al Real-time estimation of aerodynamic features for ambulatory voice biofeedback. The Journal of the Acoustical Society of America. 2015;138(1):EL14–EL19. 10.1121/1.4922364 26233054 PMC4499052

